# Recent Advances in the Role of Arid5a in Immune Diseases and Cancer

**DOI:** 10.3389/fimmu.2021.827611

**Published:** 2022-01-19

**Authors:** Kishan Kumar Nyati, Tadamitsu Kishimoto

**Affiliations:** Laboratory of Immune Regulation, World Premier International Immunology Frontier Research Center, Osaka University, Osaka, Japan

**Keywords:** Arid5a, cancer, inflammation, IL6, immune regulation, mRNA stability

## Abstract

AT-rich interactive domain 5a (Arid5a) is a nucleic acid binding protein. In this review, we highlight recent advances in the association of Arid5a with inflammation and human diseases. Arid5a is known as a protein that performs dual functions. In *in vitro* and *in vivo* studies, it was found that an inflammation-dependent increase in Arid5a expression mediates both transcriptional and post-transcriptional regulatory effects that are implicated in immune regulation and cellular homeostasis. A series of publications demonstrated that inhibiting Arid5a augmented several processes, such as preventing septic shock, experimental autoimmune encephalomyelitis, acute lung injury, invasion and metastasis, immune evasion, epithelial-to-mesenchymal transition, and the M1-like tumor-associated macrophage (TAM) to M2-like TAM transition. In addition, Arid5a controls adipogenesis and obesity in mice to maintain metabolic homeostasis. Taken together, recent progress indicates that Arid5a exhibits multifaceted, both beneficial and detrimental, roles in health and disease and suggest the relevance of Arid5a as a potential therapeutic target.

## 1 Introduction

Interleukin (IL) 6 is a pleiotropic and multi-functional cytokine (1). Many cells secrete IL6, which is transcriptionally activated by various stimuli, including the inflammatory cytokines, IL1, and tumor necrosis factor α (TNFα) (2). Uncontrolled expression of IL6 is responsible for several autoimmune inflammatory diseases and tumor growth (3). The anti-IL6 receptor antibody, Tocilizumab, has been approved by food and drug administration of the United States for the treatment of autoimmune diseases including rheumatoid arthritis (RA), juvenile idiopathic arthritis, ulcerative colitis, psoriatic arthritis, and giant cell arteritis. This antibody has been further in use to treat Castleman’s disease, polymyalgia rheumatica, and Takayasu’s arteritis. Another anti-IL6 receptor antibody, Sarilumab and an antibody to block IL6, Siltuximab are also being used to treat these diseases. Recently, these drugs (Tocilizumab and Sarilumab) have been recommended by the National Institutes of Health, U.S.A. for the patients who require supplemental oxygen, high-flow oxygen, noninvasive ventilation, or mechanical ventilation. However, blocking IL6 may lead to secondary immune responses. Therefore, IL6 expression must be strictly controlled at the transcriptional and post-transcriptional levels. The transcriptional control of *Il6* expression by transcription factors (TFs), such as nuclear factor kappa-light-chain-enhancer of activated B cells (NFκB) and CCAAT-enhancer-binding proteinβ (C/EBPβ), has been studied extensively (4, 5). However, little is known regarding the manner in which *Il6* messenger RNA (mRNA) is post-transcriptionally regulated. The regulation of cytokine mRNA stability by RNA-binding proteins (RBPs) is essential because of their roles in life-threatening human diseases. Previously, we observed that chlorpromazine (CPZ), an anti-histaminic and anti-psychotic drug, inhibits lipopolysaccharide (LPS)-induced IL6 production in macrophages, whereas other inflammatory cytokines were not affected (6). During the study of the molecular mechanism of CPZ-induced inhibition of IL6, we identified a unique molecule, AT-rich interactive domain 5a (Arid5a), which is also inhibited by CPZ. We also determined its unique function in mRNA stability by binding to the 3′ untranslated region (3’ UTR) of *Il6* mRNA. However, little is known regarding the manner in which Arid5a regulates mRNAs post-transcriptionally and whether is associated with the development of inflammatory and autoimmune diseases or cancer.

**Figure 1:**
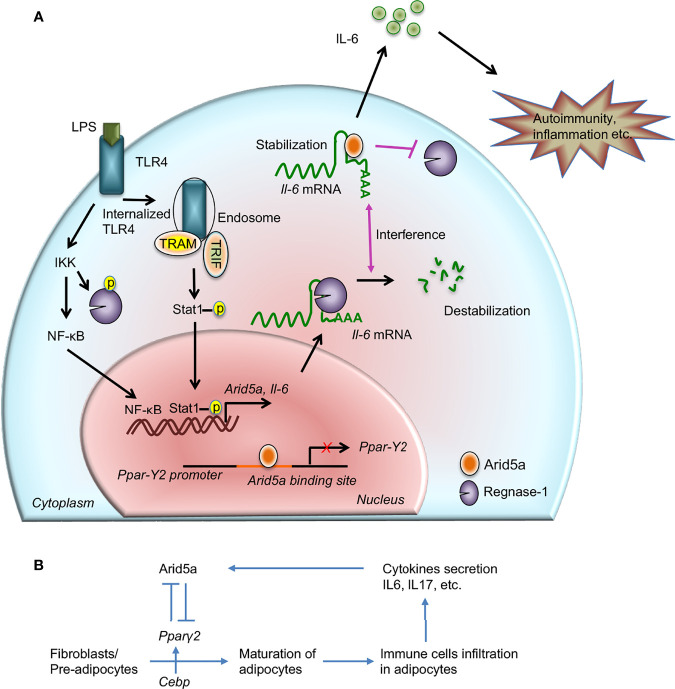
Contribution of AT-rich interactive domain 5A (Arid5a) in interleukin (IL) 6 production and disease. **(A)** The innate immune response is generated by pathogen-associated molecular patterns, which are recognized by pathogen recognition receptors and lead to expression of proinflammatory mediators. For example, toll-like receptor 4 recognizes lipopolysaccharide and activates the IKK/NFκB signaling pathway. In the early phase of TLR4 signaling, the IKK complex phosphorylates Regnase-1 and also promotes transcription of *Il6* and *Arid5a*. Regnase-1 binds to the *Il6* mRNA and degrades it. In the late phase of LPS stimulation, internalization of TLR4 or TLR4 endocytosis under TRIF signaling augments noncanonical phosphorylation of Stat1 which further induces the expression of *Arid5a*. In the nucleus, Arid5a binds to genes encoding transcription factors, such as *Pparγ2*, to inhibit their expression. Furthermore, Arid5a is shuttled to the cytoplasm under inflammatory conditions where it interferes with the destabilizing effect of Regnase-1. Arid5a is involved in *Il6* mRNA stabilization, and the resulting increase in IL6 production is associated with inflammation and autoimmune diseases. **(B)** Arid5a and *Pparγ2* in adipogenic homeostasis. C/ebp activates *Pparγ2*, and both are involved in adipogenesis. Immune cells, such as macrophages and T cells, infiltrate adipose tissues and provide a framework to regulate energy homeostasis. In adipose tissue, these cells secrete cytokines, such as IL6 and IL17, which induce *Arid5a*. Arid5a further represses *Pparγ*2 transcription in fibroblasts, thereby inhibiting adipocyte development and inhibiting adipogenesis and obesity. *“Reprinted from Frontiers in Immunology, 10, Kishan Kumar Nyati, Riddhi Girdhar Agarwal, Praveen Sharma, Tadamitsu Kishimoto, Arid5a regulation and the roles of Arid5a in the inflammatory response and disease, article 2790, copyright (December 2019), under the terms of the creative commons attribution license (CC-BY)”*, https://www.frontiersin.org/articles/10.3389/fimmu.2019.02790/full.

The AT-rich interactive domain (arid) is a nucleic acid binding domain that is evolutionarily conserved in higher eukaryotes. Its consensus sequence consists of approximately 100 amino acid residues and has a helix-turn-helix motif (7–10). Arid has also been identified in other proteins and it represents a family containing 15 members, which are important in development, differentiation, proliferation, and tissue-specific gene expression (11–13). Arid family members are widespread and are found in some protozoa, green algae, higher plants, fungi, and metazoans (14). Of these, Arid5a has gained significant interest in the field of immunology over the past decade and recently in cancer, because of its identification in the regulation of several TFs and inflammatory mRNA transcripts, including *Il6, Tbx21, Ox40, Stat3*, and *Pparγ*, through transcriptional and post-transcriptional mechanisms (15–20). Thus, Arid5a performs a dual function depending on its localization. In the resting stage of a cell, Arid5a is localized to the nucleus along with other nuclear proteins and regulates the function of transcription factors. Subsequently, it shuttles to the cytoplasm under inflammatory conditions (21–24), where it is involved in mRNA stabilization (22). During TLR4 signaling, LPS induces Arid5a expression in the early phase of stimulation through an IKK-mediated NFκB signaling mechanism (21) (**Figure 1A**). Furthermore, overexpression of NFκB subunits (p65 and c-Rel) in LPS-induced HEK293T cells increased the activity of the Arid5a promoter (21). However, another arm of the TLR4 pathway involving TRIF (Toll interleukin-1 receptor homology domain–containing adaptor inducing IFNβ) signaling has recently been shown to promote Arid5a-mediated IL6 expression during the late phase of LPS stimulation (25). Moreover, TLR4 endocytosis resulting from TRIF signaling following LPS stimulation activates non-canonical phosphorylation of STAT1 at Thr^749^, which promotes the expression of the Arid5a gene (**Figure 1A**).

Arid5a is indispensable in the development of inflammatory and autoimmune diseases, such as experimental autoimmune encephalomyelitis (EAE) (15), sepsis (17), RA (26), acute lung injury (ALI) (27), as well as breast (20), lung (28), pancreatic (PDAC) (29), colorectal (CRC) (29), and brain (30) cancers. In addition, Arid5a induces chondrocyte differentiation as a transcriptional partner of *Sox9*, which regulates chondrocyte differentiation through activation of *Col2a1*, a chondrocyte-specific gene (31). In the nucleus, Arid5a negatively regulates the transcription of the *major immediate early* (*Mie*) gene of human cytomegalovirus (HCMV) in a human embryonal carcinoma cell line (32). Furthermore, in the nucleus of fibroblasts, Arid5a suppresses transcription of *Pparγ*, a master regulator of adipogenesis, thereby inhibiting adipogenesis and obesity in mice (19), whereas increased expression of long noncoding RNA (lncRNA)-AU021063 in breast cancer cells by binding to its promoter region resulted in the regulation of invasion and metastasis (20). By discussing potential mechanisms of Arid5a and its influential roles in immunological disorders and cancer, we aim to encourage discussion on recent advances in Arid5a research. We highlight Arid5a-mediated transcriptional and post-transcriptional regulation of inflammatory molecules. We also describe the role of Arid5a in the development of inflammation and human disease.

## 2 Arid5a-Mediated Regulation of Inflammatory Genes

Gene expression is controlled at multiple checkpoints including at the gene transcription, mRNA translation, and mRNA degradation levels. TFs play a central role in regulating the transcription of a gene, whereas RBPs are known as key players in post-transcriptional regulation. Several RBPs are reported to stabilize or de-stabilize mRNAs by binding to the 3’UTR. The versatility of RNA-binding domains of RBPs and structural flexibility facilitates the control of metabolism of an array of mRNA transcripts (33). The significant role of TFs and RBPs in gene regulation has been established and perturbations in TF-gene and RBP-RNA network activities have been associated with disease development. Accumulating evidence has shown that post-transcriptional regulation controls numerous cellular mechanisms, including proliferation, differentiation, invasion, metastases, apoptosis and angiogenesis, which could lead to a cancerous phenotype (34). It is likely that the transcripts of nearly all known oncogenes and tumor suppressor genes may be controlled by mechanisms that define mRNA stability and, ultimately, competency for translation. As discussed, Arid5a contains a nucleic acid binding domain that enables it to bind to either DNA or RNA. This ability imparts a dual function to Arid5a in the stabilization of mRNAs, including *Il6* (15), *Tbx21* (encodes *Tbet*) (17), *Stat3* (16), and *Ox40* (18), and in the transcription of genes, such as *Pparγ* (19) and lncRNA-*AU020163* (20), some of which are associated with inflammation, autoimmunity, and cancer.

### 2.1 Transcriptional Regulation

In cardiovascular tissues, Arid5a binds to the N- and C-terminus of estrogen receptor (ER)-alpha and acts as an ER repressor of gene expression in the nucleus (35). It is expressed in cartilage to induce chondrocyte differentiation by interacting with the TF, *Sox9*, and regulates chondrocyte differentiation through activation of *Col2a1*, a chondrocyte-specific gene (31). Arid5a actually binds to the promoter of the *Col2a1* gene and enhances transcription of *Col2a1*, which further induces acetylation of histone 3 proteins to regulate chondrocyte differentiation in association with *Sox9*. Recently, we observed that Arid5a regulates the invasion and metastasis of breast cancer cells (20). Arid5a under IL6 signaling binds to the upstream region of a lncRNA, AU021063, and transcriptionally upregulates the expression of this noncoding RNA, which further leads to the enhanced invasion and metastasis of breast cancer cells in mice by stabilizing the tribbles homolog 3 (Trib3) protein (20). In contrast, Arid5a inhibits transcription of the *Mie* gene of HCMV by binding to multiple sites in the modulator region (31). We reported that Arid5a is an important negative regulator of energy metabolism in which *Arid5a* and *Pparγ* were dynamically counter-regulated by one another. Transcriptome data revealed that Arid5a binds to the promoter region of *Pparγ*, which is the leading cause of adipogenesis, and represses the transcription of the *Pparγ* gene (**Figure 1A**), thereby inhibiting adipogenesis and obesity in mouse fibroblasts (19) (**Figure 1B**).

### 2.2 Posttranscriptional Regulation

#### 2.2.1 Macrophages

CPZ was previously demonstrated to exert a specific inhibitory activity on IL6 production in LPS-stimulated mouse macrophages without affecting the production of other cytokines (e.g., TNFα) (6). To determine the underlying mechanism of IL6 inhibition by CPZ, we identified Arid5a, which binds to the *Il6* 3′ UTR in mouse macrophages in an *Il6* 3′ UTR RBP assay coupled with mass spectrometry (15). Further *in vitro* and *in vivo* experiments established a relationship between Arid5a protein and *Il6* mRNA suggesting that the ARID domain of Arid5a binds to the *Il6* 3′ UTR and requires *Il6* mRNA for stability (**Figure 1A**). Interestingly, Arid5a interrupts the function of Regnase-1 or Zc3h12a (15) in macrophages, which is a novel RNase that harbors a CCCH-type zinc-finger domain and a PIN-like domain. It has the ability to destabilize *Il6* mRNA by binding to the stem-loop region in the *Il6* 3′ UTR (36, 37). This indicates regulation of *Il6* mRNA through a balance between Arid5a and Regnase-1 activities (**Figure 1A**).

#### 2.2.2 Cardiac Fibroblasts

Studies describing a post-transcriptional regulatory role for Arid5a in different cell types have established it as an RBP. Cardiac fibroblasts secrete numerous cytokines and growth factors to regulate cardiac function (38, 39). Angiotensin II-stimulated cardiac fibroblasts release IL6 family proteins that facilitate the growth of cardiomyocytes (39). The β-adrenergic receptor (βAR) agonist also enhances the production of proinflammatory cytokines (40, 41), which cause heart failure. Thus, inhibition of production of proinflammatory cytokines may be a useful strategy for cardiac remodeling. A recent study suggested that β2-adrenergic stimulation induces Arid5a, which further post-transcriptionally upregulates the expression of *Il6* mRNA through the cAMP/PKA/CREB pathway in adult cardiac fibroblasts, and indicates that the β2AR/Arid5a/IL6 axis may be a therapeutic target to prevent cardiac inflammation (42).

#### 2.2.3 T Cells Through Regulation of T Cell Differentiation

**Figure 2:**
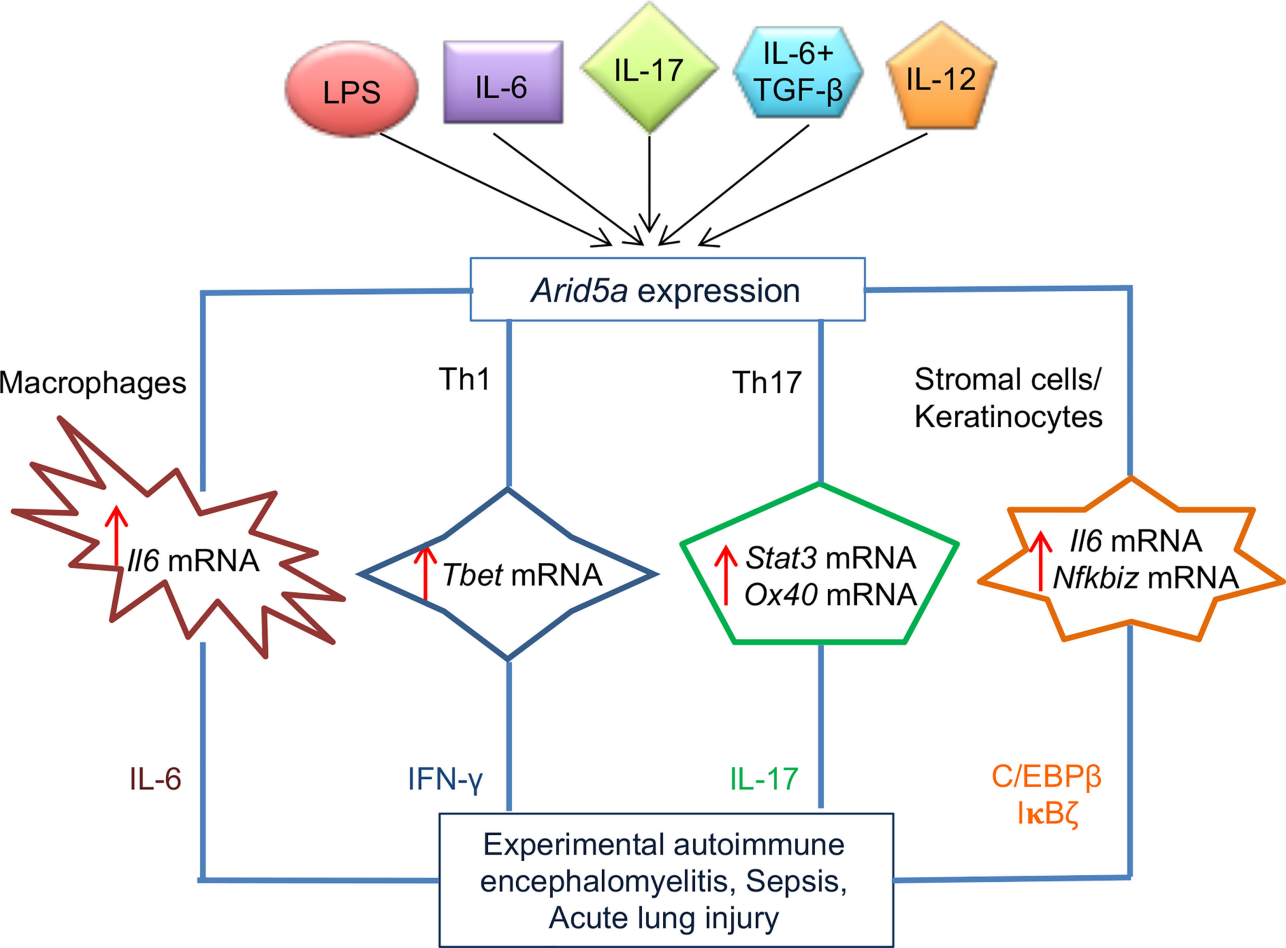
Role of AT-rich interactive domain 5A (Arid5a) in the pathogenesis of sepsis, acute lung injury (ALI), and experimental autoimmune encephalomyelitis (EAE) in mice. *Arid5a* expression is induced in many immune cells, such as macrophages, T helper (Th)1 cells, Th17 cells, stromal cells, and keratinocytes, after stimulation with lipopolysaccharide (LPS), interleukin (IL)6, IL6 + transforming growth factor (TGF)β, IL17, or IL-2. Arid5a stabilizes a variety of inflammatory mRNAs, including *Il6*, *Tbx21*, *Stat3*, *Ox40*, and *Nfkbiz*, in these cells. The stabilization of these target mRNAs results in overproduction of IL6, interferon (IFN)γ, IL17, CCAAT-enhancer-binding proteins (C/EBP)β, and inhibitor of κB (IκB)ζ, which triggers pathogenesis of sepsis, ALI, and EAE in mice. “Reprinted from Trends in Immunology, 41(3), Kishan Kumar Nyati, Mohammad Mahabub-Uz Zaman, Praveen Sharma, Tadamitsu Kishimoto, Arid5a, an RNA-binding protein in immune regulation: RNA stability, inflammation, and autoimmunity, p255–268, copyright (March 2020), with permission from Elsevier”.

Reports indicate that *Arid5a^−/−^* mice have low numbers of CD4+IL17+ T cells, or Th17 cells, or IFNγ-producing CD4+ T cells (Th1 cells) compared with wild-type mice (16, 17). This indicates a significant involvement of Arid5a in the differentiation of naive Th cells into Th1 and Th17 cells. possibly through the regulation of *Tbet* and *Stat3* mRNA stability, respectively (16) (**Figure 2**). The differentiating conditions of Th17 with TGFβ1 and IL6 in early stages of CD4+ T cells induces the rapid expression of *Arid5a*, but not at later times, which indicates a role for Arid5a in the early phase of Th17 cell development (16). *Stat3* is induced by IL6 canonical signaling in CD4+ T cells and IL6/Stat3 axis signaling contributes to the development of Th17 cells and is associated with several human diseases, such as multiple sclerosis (MS) and RA (43–46). Arid5a binds to the stem region of the *Stat3* 3′ UTR (1738–1765) through the R128 residue, where Regnase-1 also binds. Regnase-1 recognizes a unique sequence, UGU, in the loop region of the mouse *Stat3* 3′ UTR (1738-1765) (47). Furthermore, Regnase-1 reduces the luciferase reporter activity of the *Il6* 3′ UTR as mentioned previously (37), and impairs the luciferase reporter activity of the *Stat3* 3’ UTR, which is rescued by overexpression of *Arid5a* in 293T cells (16). This indicates that both Arid5a and Regnase-1 target *Stat3* (16) and indicates that Arid5a prevents Regnase-1 from binding to the *Stat3* 3′ UTR, leading to interference of Regnase-1 function (16). In addition, the population of Th17 cells was recovered following overexpression of Stat3c in *Arid5a^−/−^* CD4+ T cells under Th17-polarizing conditions, which further suggested a central role of Arid5a in Th17 cell differentiation through *Stat3* (16).

Similarly, to regulate *Stat3* and *Il6* mRNAs, Arid5a regulates the stability of other mRNAs, such as Ox40 in murine CD4+ T cells under Th17-polarizing conditions and *Tbx21* in Th1 cells (17, 18). Treatment of CD4+CD62L+ T cells from *Arid5a^−/−^* mice with actinomycin D showed a reduction in the half-life of *Ox40* mRNA (18), whereas overexpression of *Tbx21* 3’ UTR and Arid5a constructs in HEK293T Tet-off cells resulted in a significantly higher mRNA stability of *Tbx21* following doxycycline treatment (17). In these cells, Arid5a binds to the stem-loop structures of the *Ox40* and *Tbx21* mRNA transcripts, which further indicates the Arid5a recognition of stem-loop regions of the *Stat3* and *Il6* 3’ UTRs (17, 18) (**Figure 2**). Similar to Th17 cell differentiation, Th1 cell differentiation is also suppressed in *Arid5a^−/−^* mice by decreasing the half-life of *Tbx21* mRNA and leading to a reduction in IFNγ production (17) (**Figure 2**). Taken together, Arid5a controls Th1 and Th17 cell differentiation by regulating the stability of *Tbx21, Stat3*, and *Ox40* mRNAs in T cells.

Other studies have demonstrated that Arid5a binds directly to the 3′ UTRs of multiple target mRNAs and stabilizes IL17-induced mRNA transcripts, such as *Il6* and CXC chemokines (48). Furthermore, Arid5a enhances the translation of IL17-induced TFs, such as C/ebpβ and IκBζ (**Figure 2**), which attenuates the expression of the *Lcn2* gene. Moreover, Arid5a promotes IL17 signaling by stabilizing the expression of several IL17-dependent cytokine mRNA transcripts including *Il6*, *Cxcl1*, and *Cxcl5*. In additional, Arid5a induces translation of IL17-dependent TFs (*C/ebpβ* and *IκBζ*) that facilitate transactivation of the *Lcn2* promoter. Arid5a failed to activate a mutant of the C/EBP binding element and inhibiting *Arid5a* further decreased IL17-induced expression of all C/EBP protein isoforms. The expression of Arid5a is increased through a feed-forward loop in IL17-stimulated cells, which further triggers the recruitment of Arid5a to TRAF2 (23, 24, 48). Taken together, Arid5a drives Th17 signaling by regulating IL17-dependent TFs, which may represent an approach to treat IL17-dependent diseases (24).

#### 2.2.4 CRC and PDAC Cells

**Figure 3:**
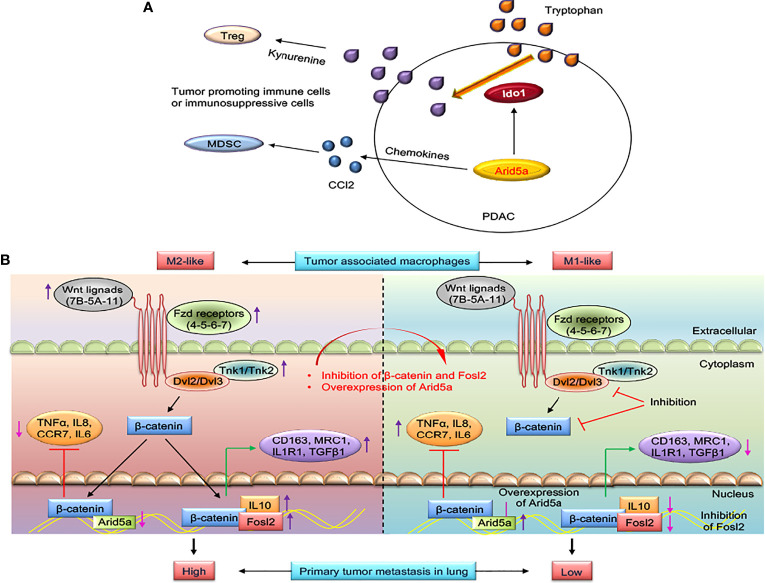
AT-rich interactive domain 5A (Arid5a)-mediated immune evasion in pancreatic cancer and reprogramming of M2-like tumor-associated macrophages (TAMs) to M1-like TAMs in lung cancer. **(A)** Epithelial-to-mesenchymal transition-transcription factors (EMT-TFs) induces Arid5a expression in mesenchymal subtypes of pancreatic cancer (PDAC) which promotes tumorigenesis by increasing immunosuppressive cells such as Ccl2-mediated recruitment of MDSCs and Ido1-dependent Tregs differentiation. Arid5a stabilizes *Ido1* and *Ccl2* mRNA transcripts leading to IDO1-mediated degradation of degrade tryptophan which ultimately reduce activation of effector T cells, but enhances Tregs differentiation. Further, CCL2 enhances recruitment of neutrophils, MDSCs, and monocytes into the tumor microenvironment facilitates tumorigenesis. **(B)** Wnt ligands (Wnt5A-7B-11), frizzled receptors (Fzd4-5-6-8-9), disheveled (Dvl2-3), and Tnk (Tnk1-2) are upregulated in M2-like TAMs, and in this way, transcriptional activation of *β-catenin* occurs. β-catenin binds to the promoter region of M2 macrophage genes such as *IL10* and to TF-activating M2 macrophage genes like *Fosl2*, *CD163*, *MRC1*, *IL1R1*, and *TGFβ1* and thus activates the M2 macrophage program. In contrast, β-catenin suppresses the M1 macrophage program by binding to TF activating M1 macrophage genes such as *Arid5a*, *TNFα*, *IL8*, *CCR7*, and *IL6*. With inhibition of *β-catenin* and *Fosl2* and overexpression of Arid5a, M2-like TAMs are reprogrammed into M1-like TAMs; thus, reactivation of antitumor immunity in the tumor microenvironment occurs to restrict primary and metastatic lung tumor growth.

Although the functions of TFs in cancer are well studied, the role of TFs in RNA-binding activities is less clear. Several RBPs that are required for complete RNA metabolism from production to degradation are mutated in cancer; however, only a few are recognized as major cancer drivers (49). Arid5a, which functions as a TF and RBP, was recently shown to be involved in cancer. Our laboratory recently reported an association of Arid5a with CRC and PDAC (29). We found that the injection of *Arid5a^−/−^* tumor cells into immunocompetent and immunodeficient mice resulted in smaller tumors in the immunocompetent mice compared with the immunodeficient mice. This prompted us to consider the possibility of an Arid5a-mediated immunosuppressive effect in the tumor microenvironment (TME). This hypothesis is further supported by the observation of a higher frequency of effector CD8+ T cells, whereas lower numbers of immunosuppressive cells, such as regulatory T cells (Tregs) and myeloid-derived suppressor cells (MDSCs), were present in *Arid5a^−/−^* tumors compared with wild-type tumors. Subsequently, a series of studied revealed that Arid5a induces the expression of *Ido1* and *Ccl2* by increasing the half-lives of their mRNA transcripts. IDO1, a rate-limiting metabolic enzyme, degrades tryptophan, which ultimately reduces the activation of effector T cells, but enhances Treg differentiation (50, 51). The inhibition of *Ido1* resulted in a T cell-dependent anti-tumor response in mice (52, 53) and CCL2 was involved in the recruitment of neutrophils, MDSCs, and monocytes into the TME, which was associated to tumorigenesis (54, 55). Collectively, Arid5a mediates immune evasion in PDAC and CRC models by inducing an Ido1-dependent local immunosuppressive TME and CCL2-mediated MDSC recruitment (**Figure 3A**). An in-depth understanding of the role of Arid5a in these cancer models will contribute to the development of prognostic and response biomarkers and may lead to new therapeutic strategies.

## 3 Linking Arid5a-Mediated Transcriptional and Post-Transcriptional Regulatory Mechanisms

An earlier study demonstrated the time-dependent subcellular localization of a substantial fraction of *Arid5a* from the nucleus to the cytoplasm under inflammatory conditions, such as LPS exposure (22). The study has also found that nuclear *Il6* mRNA was increased in the early phase of LPS stimulation, whereas cytoplasmic *Il6* mRNA was increased in macrophages at later time points. Furthermore, RNA immunoprecipitation assays using anti-Arid5a antibodies coupled with qPCR revealed a 3.9-fold increase in *Il6* mRNA bound to cytoplasmic Arid5a and a 2.6-fold increase in nuclear *Arid5a* following LPS stimulation compared with unstimulated control cells. These data indicate that both nuclear and cytoplasmic Arid5a have the ability to bind to inflammatory mRNAs, such as *Il6*, even after LPS stimulation. Thus, Arid5a plays different roles in the regulation of mRNAs depending on its subcellular (nuclear or cytoplasmic) localization. It is therefore likely that nuclear and cytoplasmic Arid5a perform dual functions simultaneously which are associated with its transcriptional and post-transcriptional regulatory activities. As mentioned, the dual function of Arid5a depends on time, inflammation, and disease conditions. However, a correlation between these two mechanisms has not been established. These mechanisms warrant further study with respect to the role of Arid5a in reducing inflammation by inhibiting Arid5a-associated transcriptional regulation in the nucleus and post-transcriptional regulation of inflammatory genes in the cytoplasm.

## 4 Arid5a-Associated Inflammatory and Autoimmune Diseases

### 4.1 ALI by Regulating IL6 and Reactive Oxygen Species Production

ALI with acute respiratory failure causes acute inflammation in the lungs and is responsible for a high mortality worldwide (56, 57). No treatment exists other than maintaining patients on mechanical ventilation during ALI. Bleomycin-induced pulmonary fibrosis and respiratory inflammation in mice mimic ALI, in which IL6 is abnormally upregulated (58). *Il6*-deficiency in mice improved recovery from lung fibrosis and ALI following bleomycin administration (59), but the underlying mechanism remains elusive. In preliminary studies, bleomycin-treated *Arid5a^−/−^* mice exhibited less reactive oxygen species (ROS) production, lung inflammation, and were found to be resistant to ALI (27). The reduction in ROS affected oxidized 1-palmitoyl-2-arachidonoyl-snglycero-3-phosphocholine (OxPAPC) production. This attenuated IL6 production is because of the absence of post-transcriptional regulation by Arid5a in *Arid5a*-deficient mice (27) (**Figure 2**). However, further studies are needed to shed light on Arid5a-mediated regulation of ROS production during ALI.

### 4.2 Septic Shock by Controlling *Tbet*-Mediated IFNγ Production and Th1 Cell Differentiation

Endotoxin or LPS present in the Gram-negative bacteria is the primary cause of septic shock, which is defined as a severe systemic inflammatory condition and is one of the bases for “cytokine release syndrome”. Several proinflammatory cytokines, including IFNγ and IL6, play a central role in the development of septic shock and activate T cells and NK cells by binding to specific cell receptors (60). The transcription factor, *Tbet*, enhances IFNγ production in T cells and directs Th1 cell differentiation (61, 62). *Tbet*-deficient mice are unable to differentiate naive T cells into Th1 cells and produce low amount of IFNγ (62). Therefore, disruption of *Tbet* expression results in various Th1 cells-associated diseases and provides evidence of a role for Tbet regulation in the innate immune response (63, 64). The inhibition of Arid5a in LPS-treated mice reduces the IL6 levels in serum (15) and results in the reduced frequency of Th1 and Th17 cells in *Arid5a^−/−^* mice (16, 17). In a study of Arid5a in immune regulation, Zaman et al. (17) observed that *Arid5a*-deficient mice are extremely resistant to LPS-administered endotoxic shock. They also found an association with reduced levels of proinflammatory cytokines, including IFNγ and IL6 compared with wild-type counterparts. Mechanistically, Arid5a targets *Tbet* mRNA and increases the expression of *Tbet* mRNA by binding to the 3’ UTR, thus attenuating the production of IFNγ. Moreover, Arid5a deficiency decreased the stability of *Tbet* mRNA due to a lack of Arid5a-mediated post-transcriptional regulation, which resulted in reduced levels of IFNγ in T cells during Th1 cell polarization (**Figure 2**). Furthermore, *Arid5a^−/−^* mice were resistant to *Propionibacterium acnes*-primed LPS exposure, indicating that Arid5a regulates IFNγ and IL6 by increasing the stability of *Tbet* and *Il6* mRNA transcripts, respectively. The synergistic amplification of several cytokines likely occurs which eventually results in septic shock. Therefore, inhibiting Arid5a expression may represent a useful approach to limit some of pathologies associated with septic shock.

### 4.3 Obesity by Inhibiting Transcription of *Pparγ*

Obesity can be inhibited by limiting adipogenesis, which is associated with IL6 expression in mice (19, 65). *Arid5a* knockout mice exhibit low levels of IL6 because of the absence of Arid5a-mediated *Il-6* mRNA post-transcriptional upregulation (15). IL6 also induces *Arid5a* expression (20, 21). This increases the probability of Arid5a involvement in IL6 inhibition of adipogenesis and obesity. We reported that IL6-stimulation in *Arid5a^−/−^* 3T3-L1 adipocytes inhibited the differentiation of adipocytes (19) and increased the possibility of a role for Arid5a in inhibiting adipocyte differentiation. *Il6*-deficiency in mice resulted in higher adipogenesis and obesity, which result from insufficient Arid5a induction. Following stimulation by IL6, Arid5a further represses *Pparγ* expression, which ameliorates adipogenesis and obesity. Pparγ is a major regulator of adipogenesis and obesity, and its increased expression is responsible for weight gain in animals and humans (66). *Pparγ2* was found to be upregulated in *Arid5a^−/−^* 3T3-L1 cells and mouse embryonic fibroblasts (MEFs) at early time points of adipocyte differentiation (19). An immunofluorescence assay revealed that in the early phase of adipocyte differentiation, *Pparγ2* promoter activity was higher compared with normal conditions in 3T3-L1 cells; however, increased expression of Arid5a, following transfection of an Arid5a expression vector, significantly reduced *Pparγ2* promoter activity. The underlying *in vitro* mechanism may involve the binding of Arid5a to the *Pparγ2* promoter, which inhibited *Pparγ2* transcription (19). This binding further impaired the interaction of Pparγ2 with C/EBP, which is another regulator of adipogenesis and eventually limits the adipogenesis process in mice (**Figure 1B**). However, these findings draw further attention to the role of Arid5a in regulating adipogenesis and obesity and provide further insight into the coordinated activity of Arid5a and other targets, such as Sox9, that are involved in these biological processes (67).

### 4.4 EAE by Coordination of *IL6*, *Stat3*, and *Ox40* mRNAs Regulation and Th17 Cell Differentiation

EAE is an animal model of MS, which has been associated with a pathogenic role for Th17 cells and abnormal levels of IL6 and Stat3. Since Arid5a regulates *Il6* and *Stat3* expression in immune and nonimmune cells, *Arid5a* deficiency protects mice from developing EAE, in which IL17-producing T cells are markedly decreased (15). Furthermore, Stat3 activation is required for the differentiation of Th17 cells (68–70); however, loss of *Arid5a* in T cells of *Arid5a*-deficient mice reduces the expression of *Stat3* and *Stat3*-regulated genes, such as *Rora*, *Rorc*, and *Batf*, resulting in a lower number of Th17 cells (16). Inflammatory CD4+ T cells from the spinal cord of EAE mice express Ox40 (71, 72), which further facilitates IL17 expression. CD4+ T cells from *Arid5a^−/−^* mice under Th17 polarizing conditions exhibit decreased expression of *Ox40* and are unable to induce IL17 expression. In addition, *Stat3* deficiency in CD4+CD62L+ T cells had lower expression of *Ox40* under Th17-polarizing conditions. Stat3 increases the survival of CD4+ T cells through *Ox40*, *FasL*, and *Bcl2* expression in wild-type mice compared with *Stat3^−/−^* mice (73). Furthermore, Arid5a targets a stem loop in the *Ox40* 3’ UTR to enhance mRNA stability. Thus, Arid5a and Stat3 are involved in the regulation of *Ox40* expression. Furthermore, adoptive transfer of *Arid5a*-deficient encephalitogenic CD4+ T cells impaired EAE through the Arid5a/Ox40 axis in CD4+ T cells, and is required for IL17 production and EAE development (**Figure 2**). Collectively, Stat3 and Ox40 are regulated by Arid5a in CD4+ T cells in which IL6 is equally important. The coordinated activities of these molecules may regulate Th17 signaling.

## 5 Association of Arid5a in Cancer

**Figure 4:**
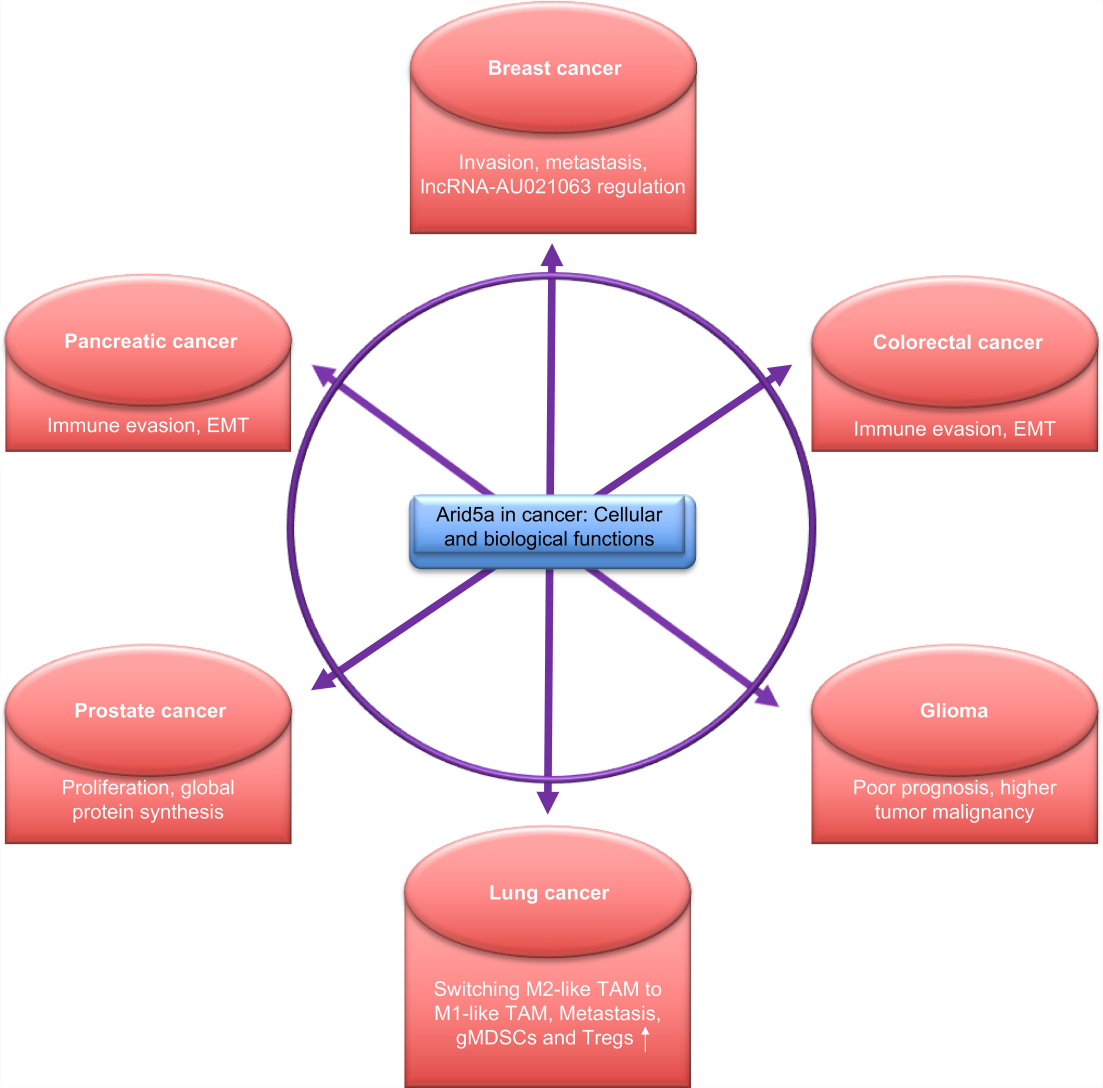
Functions of AT-rich interactive domain 5A (Arid5a) in cancer. Arid5a regulates various cellular processes *via* transcriptional and post-transcriptional modifications and is associated with the regulation of various cellular activities, as depicted above.

An association of Arid5a-regulated molecules, such as *IL6*, *Tbet*, *Stat3*, *Ox40*, and *Pparγ*, has been widely studied in various cancers and tumor models. Therefore, it is likely that Arid5a contributes to many processes in cancer cells. Interestingly, recent studies have reported a role for Arid5a in many cancers including glioma, breast, pancreatic, lung, and colorectal cancer (**Figure 4**).

### 5.1 Arid5a-Mediated Wnt Signaling in Lung Cancer Reprograms M1-Like Tumor-Associated Macrophages (TAMs) Into M2-Like TAMs

Lung cancer is responsible for the majority of cancer-associated deaths in men and women globally (74). The 5-year survival rate for metastatic lung cancer is reported to be approximately 5%, which is considerably lower compared with other cancers (75). The majority of lung cancer cases cannot be treated with surgery. Therefore, chemotherapy and radiotherapy are used to treat these cases; however, side effects of these therapies disrupt normal tissue homeostasis. Even with immune checkpoint blockade therapies targeting cytotoxic T lymphocyte-associated protein 4 (CTLA-4) and PD-1 in are unable to achieve the expected outcomes because of significant toxicity (76–79). Thus, studies are required to identify new treatment options, which may be achieved through an understanding of other tumor-associated cells in the lung.

A central role of β-catenin-mediated Wnt signaling and transcription of fos-like antigen 2 (*Fosl2)* and *Arid5a* in the transition of tumor-inhibiting M1-like TAMs into tumor-promoting M2-like TAMs has recently been identified, which suggests a role for TAM-specific signaling in immune evasion in lung cancer (28) (**Figure 3B**). Polarization of macrophages (M1/M2) upon stimulation by LPS/IFNγ or IL4 modifies the extrinsic and intrinsic properties of macrophages (80) and enables them to act differently in the TME (81, 82). Alternatively, transcriptional activation *Fosl2* and *Arid5a* inhibition by β-catenin in M2-like TAMs and partially regulation of macrophage gene expression by *Tnf* and *Ccr2* results in the suppression of lung tumorigenesis and metastasis in lung cancer models developed using *in vitro*-trained TAMs and *ex vivo*-cultured TAMs isolated from mouse and human lung tumors (**Figure 3B**). Furthermore, high expression of *β-catenin* and *Fosl2* and low expression of *Arid5a* were found in a transcriptome analysis of lung cancer cases and were correlated with poor prognosis. This study may provide a strategy for developing new treatment options by targeting β-catenin-dependent gene regulation in M2-like TAMs in lung cancer (28).

### 5.2 Regulation of Invasion and Metastasis by Arid5a in Breast Cancer

Breast cancer-associated deaths in women are estimated a number of 280,000 new cases annually in the United States (83). Despite advances in diagnosis and treatment, the prevention of tumor recurrence and metastasis are the major challenges to achieving complete remission in breast cancer. The clinical efficacy of gene-targeted therapies is also limited; therefore, there is a need to identify new biomarkers and therapeutic targets.

Abnormal IL6 concentrations in breast cancer is associated with the poor survival of patients, cancer progression, invasion, and metastasis, and is increased with tumor grade (84–86). In addition, lncRNAs regulate gene or protein expression at the transcriptional, post-transcriptional, and post-translational levels (87), and were associated with MAPK signaling pathways in breast cancer (88, 89). IL6 signaling, function, and the development of several disorders resulting from IL6 dysregulation have been implicated in the regulation of lncRNAs. The upregulation of lncRNA-*UICC* and IL6 signaling in cervical cancer promotes tumorigenesis and metastasis in cervical cancer (90). Analysis of transcriptomic and genomic data revealed that the expression of lncRNA-*AU021063* was significantly upregulated in IL6-stimulated 4T1 breast cancer cells. *Arid5a^−/−^* 4T1 cells exhibited lower expression of lncRNA-*AU021063* in a fluorescent *in situ* hybridization assay, and was restored following ectopic overexpression of Arid5a in these cells under IL6 signaling. Notably, Arid5a regulates genes and mRNAs at the transcriptional and post-transcriptional level, resulting in the development of inflammation and disease (15, 17, 19). ChIP sequencing coupled with qPCR verified the binding of the Arid5a promoter region to lncRNA-*AU021063*, which coordinates with Arid5a to increase the transcription of lncRNA-*AU021063*. In addition, *Arid5a* deficiency in 4T1 cells decreased the invasion and metastasis of breast cancer cells in *in vitro* and *in vivo* studies (20). LncRNA-*AU021063* contributed to the regulation of Trib3 protein stability. Trib3 is induced by IL6 in breast cancer cells (91), promotes the malignant behavior of ovarian cancer cells through Mek-Erk signaling (92), and induce cell migration, invasion, and metastasis of liver cancer cells (93). Overexpression of lncRNA-*AU021063 in* breast cancer cells prolonged the activation of the Mek1/2 and Erk1/2 kinases and increased the production of Trib3 protein under IL6 signaling. Furthermore, inhibiting *Trib3* expression in 4T1 cells reduced invasion and metastasis of IL6-treated 4T1 cells. This effect could not be restored by overexpressing lncRNA-*AU021063*, which suggests that Trib3 upregulation mediated by lncRNA-*AU021063* contributes to the invasion and metastasis of breast cancer cells during IL6/Arid5a signaling. This study may lead to strategies for using Arid5a and lncRNA for the treatment of breast cancer.

A database mining study recently assessed the expression of Arid family members including Arid5a based on The Cancer Genome Atlas (TCGA) data and also evaluated the prognostic value of each member in breast cancer patients (94). The mRNA expression of *Arid3a, Arid3b, Arid4b, Jarid1b, Jarid1c*, and *Jarid2* was higher, whereas *Arid1b, Arid3c, Arid4a, Arid5a, Arid5b*, and *Jarid1a* mRNA expression was lower in tumor tissues compared with controls. Furthermore, the expression of *Arid1a, Arid1b, Arid2, Arid3a, Arid4a*, and *Arid4b* in non-luminal subtypes of breast cancer was lower compared with that in luminal subtypes. Conversely, *Arid3c, Arid5a*, and *Jarid2* mRNA expression was higher in non-luminal subtypes of breast cancer. A Kaplan-Meier analysis revealed that lower expression of *Arid5a* mRNA was associated with poor overall survival in the luminal type and all breast cancer patients. Based on an analysis of online datasets, Arid5a is considered a good candidate for tumor treatment in breast cancer (94); however, it will be necessity to conduct further studies to provide more experimental evidence.

### 5.3 Immune Evasion by Arid5a in CRC and PDAC

Immunotherapy has become a better option for the treatment of advanced cancers (95). Although, many patients are resistant to immune checkpoint blockade (ICB) (96), it is useful for the treatment of metastatic tumors, and results in sustained tumor regression. The plasticity and heterogenicity of tumors along with the TME contributes to ICB resistance and immune evasion. Immune evasion is a hallmark of cancer activated by an immune checkpoint pathway, such as PD1-PDL1 signaling, or an increase in immunosuppressive factors (97). CRC and PDAC are considered cold tumors and are non-immunogenic because they have low numbers of tumor infiltrating cells (98, 99). The tumor-intrinsic mesenchymal phenotype of CRC and PDAC expedite immune evasion by interacting with stromal immune cells in the TME (96). The epithelial-to-mesenchymal transition (EMT) process enhances invasion and migration, and the immunosuppressive properties of tumor cells; however, a precise molecular mechanism has not been defined.

Recently, we demonstrated an association of Arid5a with the mesenchymal subtypes, CMS4 and QM, and immune evasion in CRC and PDAC (29). In addition, Arid5a mediates immune evasion by recruiting immunosuppressive cells, such as MDSCs and Tregs, and decreasing antitumor effector T cell recruitment and activation in the TME (29) (**Figure 3A**). *Arid5a^−/−^* tumor cells produced smaller tumors in immunocompetent mice compared with immunodeficient mice, which further supports a role for Arid5a in immune evasion and immunosuppression in the TME. During EMT in human and murine pancreatic cancer lines, higher expression of Arid5a was observed, which is due to induction by EMT-TFs, but not by *Stat3* in mesenchymal cells of CRC and PDAC. High expression of Arid5a further correlated positively with TGFβ1 and IL6 levels. As mention above, *Ido1* and *Ccl2* mRNAs are stabilized by Arid5a. Therefore, increased IDO1 activity facilitates immunosuppression by metabolizing tryptophan and results in deceased effector T cell proliferation and promotes Treg differentiation, and CCL2 recruits MDSCs to the tumor more efficiently (29) (**Figure 3A**). However, the molecular mechanisms underlying the regulation of Arid5a in mesenchymal tumors remain unclear and these mechanisms will be interest in future studies, especially in targeting Arid5a for tumor immunotherapy.

### 5.4 Arid5a Regulation of Proliferation and Global Protein Synthesis in Prostate Cancer

Prostate cancer (PCa) is a common neoplasm in the urinary tract of men and results in approximately 260,000 deaths annually worldwide (83). PCa is considered a global health issue because of the increasing number of prostate cancer cases in developing countries (100). Serum prostate-specific antigen (PSA) levels and pathological stage are the major diagnostic criteria for PCa. Tumor recurrence and metastasis following surgery is observed in 30% of PCa patients (101). Some cases develop aggressive castration-resistant PCa (102, 103) after androgen deprivation therapy. Therefore, the identification of new molecular markers is necessary to improve the prognosis and diagnosis of PCa patients. IL6 is highly expressed in tumors and modulates progression by affecting proliferation, apoptosis, angiogenesis, and differentiation (104, 105). IL6 levels are positively correlated with the progression of hormone-refractory and metastatic PCa patients (106–108). IL6 induces androgen-independent cell growth *in vitro* and *in vivo* (109–112). Interestingly, a recent analysis of microarray datasets from independent PCa cohorts revealed a significant correlation between the expression of *Arid5a* and *Il6* and further identified the transcriptional upregulation of *Il6* by Arid5a in human PCa cell lines, suggesting a complementary role of Arid5a in the regulation of *Il6* mRNA stability (113).

The inhibition of *Arid5a* expression in LNCaP PCa cells reduced global protein synthesis and resulted in lowering proliferation despite dihydrotestosterone (DHT)-stimulation (114). Inhibition of *Arid5a* facilitated cell cycle arrest at the G1 phase in DHT-stimulated cells and further decreased DHT-induced lipid accumulation. Several biological processes, such as cell survival, proliferation, and cell cycle regulatory proteins, such as HIF1a, cyclin D1, and cyclin D3, were attenuated following inhibition of *Arid5a* expression, but it did not affect mRNA expression which indicates a translational and/or post-translational role for Arid5a in protein regulation. In LNCaP cells, *Arid5a* deficiency augmented the phosphorylation of eukaryotic translation initiation factor 2a (eIF2a) by activating general control nonderepressible 2 kinase (GCN2) and RNA-dependent protein kinase (PKR)-like endoplasmic reticulum kinase (PERK) (114). These findings indicate a role for Arid5a in the regulation of proliferation and global protein synthesis in PCa; however, further studies are needed to determine the molecular mechanism of Arid5a-mediated regulation of GCN2 and PERK kinases, tumorigenesis, and tumor progression in PCa.

### 5.5 Predicting the Diagnostic and Prognostic Significance of Arid5a in Glioma

Glioma is an intracranial malignant primary tumor, which accounts for nearly 27% of central nervous system (CNS) tumors (115). Morbidity, mortality, and recurrence rates are higher in gliomas compared with other CNS tumors (116, 117). Grade 2 and 3 gliomas are considered low-grade gliomas (LGGs) and have characteristics of slow growth and low malignancy, whereas grade 4 glioma, known as glioblastoma multiform (GBM), is an invasive tumor with high recurrence and mortality (118, 119). Because of this, the successful treatment of gliomas is difficult (116) despite advanced treatment options such as surgical resection, postoperative adjuvant radiotherapy, and temozolomide-based chemotherapy. The survival of treated-glioma patients is limited because of the poor prognosis and high recurrence rate (120, 121). Next-generation gene sequencing has identified several molecular markers in gliomas as alternative options for diagnosis and improved prognosis. Two most of these biomarkers include isocitrate dehydrogenase (IDH) mutation and 1p/19q combined deletion, which have been widely used in clinical diagnosis and therapy (122). Therefore, the identification of biomarkers has attracted the interest of researchers and clinicians for determining cancer risk, improving prognosis, and predicting tumor recurrence.

A recent study determined the diagnostic and prognostic significance of Arid5a in gliomas and discovered a possible biological function for Arid5a (30). Low grade gliomas and GBM datasets were analyzed using online database, such as TCGA, and inversely correlated high expression of *Arid5a* with the prognosis of all stages of gliomas. Moreover, high expression of Arid5a is likely a marker of poor prognosis in glioma. In contrast, low expression of *Arid5a* was associated with poor prognosis of lung cancer in a previous study (28). Based on datasets from the Chinese Glioma Genome Atlas (CGGA) and TCGA RNA sequencing, as well as clinical and molecular characterization, the expression of *Arid5a* was increased with higher glioma tumor grade. In addition, *Arid5a* expression was higher in *IDH*-wild-type gliomas compared with *IDH*-mutant gliomas and in gliomas without 1p/19q co-deletion compared with 1p/19q co-deletion in gliomas. Earlier studies reported that wild-type *IDH* lacks 1p/19q co-deletion and higher tumor grade was associated with poor prognosis in glioma (123, 124). Another study reported high expression of Arid5a expression is associated with complex tumor malignancy (30), suggesting that Arid5a may be a useful marker for the degree of tumor malignancy.

The analysis of glioma samples in the TCGA datasets revealed enrichment of apoptosis, cytokine-cytokine receptor interactions, JAK-STAT signaling, leukocyte transendothelial migration, and toll-like receptor signaling in the high *Arid5a* expression group. In addition, cell adhesion molecules, ECM receptor interactions, JAK-STAT signaling, leukocyte transendothelial migration, and p53 signaling were also enriched in the high *Arid5a* expression group of gliomas from CGGA datasets by gene set enrichment analysis. Arid5a regulates *Il6* and *Stat3* mRNAs (15, 16, 23), which are further associated with the modulation of p53 gene expression and protein degradation (125, 126). Thus, Arid5a may affect the occurrence, development, and clinical prognosis of glioma by regulating the p53 and JAK-STAT signaling pathways in glioma patients, as well as cell apoptosis. Also, high levels of Arid5a expression may regulate the immune response in glioma. A gene ontology analysis suggested a correlation of inflammatory response, immune response, and IFNγ-mediated signaling pathway with higher *Arid5a* expression in glioma, which may further reveal mechanisms that affect the growth, and proliferation of cancer cells. The dual nature of IFNγ in cancer may increase tumor growth by facilitating an immunosuppressive TME (127). In contrast, it involves the suppression of angiogenesis and enhanced immunogenicity in tumors (128). Furthermore, Arid5a increases *Tbet*-meditated IFNγ production in CD4+ T cells (17). Therefore, it is likely that Arid5a modulates the IFNγ-mediated signaling pathway to support TME. However, the role of Arid5a in glioma has been primarily based on database mining; therefore, comprehensive studies utilizing cell culture, animal models, and/or patient samples is needed to confirm a regulatory function of Arid5a in glioma.

## 6 Arid5a Implies Non-Canonical IL6 Signaling in Cancer

Previous studies of Arid5a have indicated an association of Arid5a with canonical IL6 signaling. IL6 induces Arid5a mRNA expression in macrophages and MEFs (15, 21). Furthermore, Arid5a expression is inhibited in *Il6*-knockdown MEFs activated by LPS exposure (21), although Arid5a mRNA expression cannot be induced in *Stat3*-knockdown MEFs, despite LPS stimulation. These findings suggest that Stat3 under conditions of canonical IL6 signaling plays an important role in *Arid5a* expression. Further studies revealed that phosphorylated Stat3 binds to the promoter region of *Arid5a*, thus increasing its expression. Taken together, IL6-Stat3 signaling has been considered to act synergistically with LPS signaling to induce Arid5a expression in MEFs, suggesting that IL6 regulates *Arid5a* expression through a positive feedback loop that is regulated by TLR4 signaling (21). Interestingly, Arid5a was subsequently found to post-transcriptionally regulate *Stat3* mRNA in Th17 cells (16). Arid5a binds to the stem structure of the *Stat3* 3’ UTR to stabilize the transcript. Stat3 plays a key role in regulating the fate of naive CD4^+^ T cells (129, 130), and in *Arid5a*-deficient mice, the rate at which naive CD4^+^ T cells differentiate into Th17 cells is reduced because of the lower level of IL17-producing T cells and hence the relatively low expression of *IL17A*. Accordingly, the level of Stat3 in *Arid5a^−/−^* T cells contributes to the impairment of Th17 cell differentiation. In contrast, as discussed earlier, we demonstrated a role for Arid5a in immune evasion by augmenting tryptophan metabolism and chemokine expression in cancer (29). Moreover, *Arid5a* expression was associated with the mesenchymal phenotypes of CRC and PDAC, and Arid5a is involved in immune evasion by promoting tumor infiltration by MDSCs and Tregs, and by suppressing the recruitment and activation of antitumor effector T cells (**Figure 3A**). The activation of the IL6/Jak/Stat3 signaling pathway upregulates EMT-TFs, such as *Zeb1*, *Zeb2*, *Snai1*, *Snai2*, *Twist1*, *Twist2*, and *Stat3*, through which metastasis is enhanced through the induction of EMT (131). Our data also suggest the involvement of Arid5a in inducing the mesenchymal properties of EMT cell lines, such as KPC. However, analysis of PDAC RNAseq data from TCGA and KPC RNA seq revealed that Arid5a expression is significantly correlated with the expression of cytokines associated with EMT induction, such as TGFβ1 and IL6, and of representative EMT-TFs, but not *Stat3* (29). This indicates a diversity signaling pathways in cancer models, which are more likely to be different from signaling in MEFs, and other immune cells, such as Th17 and macrophages.

## 7 Therapeutic Approaches Targeting Arid5a

The inflammatory cytokines such as TNFα and IL1β are produced by the immune cells in response to pathogen attacks on humans. This generated immune response sometimes exacerbates the expression of cytokines, such as IL6, which was observed during inflammation and other disease conditions, including sepsis and influenza (132, 133). The excessive production of IL6 could be dangerous. Blocking or inhibiting IL6 may be a useful treatment in the prevention of IL6-regulated diseases (134). However, an *in vivo* study suggested that knocking out IL6 in mice was not shown recovery from sepsis (135) owing to the role of IL6 in host defense mechanism from pathogens and tissue damage (136). Therefore, it is likely that the complete elimination of IL6 can be lethal, and it calls for a substitute approach to control IL-6. Arid5a as discussed increases the expression of inflammatory mRNAs including IL6 by increasing their half-life. Targeting Arid5a may limit the production of these inflammatory mediators. No drug designs have been established so far those target endogenous proteins. Cytosolic antibodies against Trim21 that linked to an Fc receptor were observed in degradation of their analogous endogenous target proteins *via* ubiquitin-proteasome system raising hope to exploit a similar approach to target and degrade endogenous proteins such as Arid5a with antibodies. It could pose to be a potent therapeutic option to balance the action of not only IL6 but also controls inflammation.

Earlier studies have shown Arid5a is able to enhance the production of IFNγ by stabilizing T-bet mRNA (17, 23). Also, Zhou et al. have recently reported upregulation of Arid5a in glioma is positively correlated with inflammation, immune response, IFNγ-mediated signaling, and apoptosis which affect the proliferation and growth of cancer cells. IFNγ has a dual function in cancer immunology. It suppresses proliferation and induces the expression of class I MHC on cancer cells that results in enhancement of immunogenicity, and suppression of angiogenesis in tumors (128). In contrast, IFNγ stimulation can increase tumor growth and accelerate the formation of 5tumor immunosuppressive microenvironment (127). Therefore, controlling IFNγ signaling further aids in the regulation of TME *via* Arid5a.

Reported that JAK-STAT signaling is critically involved in the regulation of glioma cell survival, growth, and invasion and is known as one of the potential targets for gene therapy (137, 138). As mentioned, Arid5a regulates *Il6* and *Stat3* mRNAs (15, 16, 23), which are further associated with the modulation of p53 gene expression and protein degradation (125, 126). This suggests that Arid5a may be associated with the JAK-STAT and p53 signaling in tumorigenesis. Moreover, Arid5a may affect the occurrence, development, and clinical prognosis of glioma by regulating the p53 and JAK-STAT signaling pathways in glioma patients, as well as cell apoptosis. In addition, IL-6 promotes Arid5a expression through Stat3 activation (15). Furthermore, Arid5a stabilizes *Il6* and *Stat3* mRNAs after stimulation by IL6 *via* a feedback loop, which involves in the pathogenesis of inflammatory autoimmune diseases (15, 16). It is also reported that immature dendritic cells accelerate tumor immune tolerance and promote tumor growth (139). There are other reports showing activation of Stat3 by IL6 can inhibit the maturation and activation of dendritic cells (140), while Arid5a functions are associated with IL6/STAT3 signaling (24). Therefore, it is likely that Arid5a may be implicated with the regulation of the function of dendritic cells that can also be disrupted by inhibiting one or more of its downstream targets. For example, Stat3 inhibitor was shown reductions in inflammation and tumor development (141), which may prompt us to the application of a similar strategy against inflammatory diseases where IL6 is overproduced, such as sepsis and EAE. Several stimuli IL1β, IFNγ, LPS, and OxPAPC (15, 16, 21, 27) have been shown to induce expression of Arid5a which results in IL6 overproduction; therefore, there may be an alternative strategy to suppress Arid5a expression by inhibiting these stimuli would ultimately reduce the IL6 expression and associated diseases. Finally, in-depth research of the expression and function of Arid5a not only in immune-related diseases but also in cancers can provide accurate diagnostic values and reliable prognostic pieces of evidence, and may thus improve the clinical therapeutics of these diseases.

## 8 Concluding Remarks and Future Directions

The association of Arid5a with inflammation and autoimmune diseases and a variety of cancers has been revealed. The mechanisms underlying these diseases may occur through Arid5a-mediated transcriptional and post-transcriptional regulation of various genes and mRNA transcripts. For example, Arid5a binds to the promoter of *Pparγ* to suppress transcription, which leads to inhibition of adipogenesis and obesity and to lncRNA-*AU021063* to augment invasion and metastasis in breast cancer. It also binds to the 3ʹ UTR of the *Il6*, *Stat3, and Tbet* transcripts to enhance stability, which further exacerbates the pathogenesis of EAE and septic shock. This demonstrates the dual function of the Arid5a protein to bind both DNA and RNA to facilitate the pathogenesis of inflammatory and autoimmune diseases and cancer.

Whereas the *Il6* gene is transcriptionally upregulated by Arid5a in prostate cancer cells, Arid5a increases the half-life of *Il6* mRNA at the post-transcriptional level in immune cells, such as macrophages, which further suggests a cell-specific function of Arid5a. It is likely that IL6 and the diseases in which IL6 is overproduced may be regulated, in part, by Arid5a, and deserves further study. Nonetheless, many of the Arid5a targets identified function as TFs, such as *Tbet* and *Stat3*, Ox40 as a co-stimulatory receptor in T cell activation, or IL6 as a cytokine signal. This increases the possibility of the existence of secondary targets that have yet to be identified, although their significance is poorly understood. Certainly, many other such targets will likely be discovered in the future.

Arid5a exhibits multifactorial functions, both beneficial and detrimental, in different disease environments. Some of these involve regulating the innate and adaptive immune response, modulating cell proliferation, Th1 and Th17 cells differentiation, stabilizing mRNAs, regulating TFs, and maintaining cellular homeostasis. Thus, further studies of the Arid5a protein and its interaction with its target molecules, and revealing a relationship between transcriptional and post-transcriptional mechanism mediated by Arid5a, will provide insight into the role of Arid5a in diseases and improve therapeutic and diagnostic methods. Nonetheless, Arid5a controls several biological processes in cancer such as invasion, metastasis, reprogramming M2-like TAMs to M1-like TAMs, immune suppression/immune evasion in the TME, and regulation of lncRNAs. This suggests that Arid5a represents a therapeutic target in cancer. However, an in-depth understanding of Arid5a in the biological processes of cancer and associated TME requires further study. Although Arid5a has emerged as a new target for tumors, inflammation, and immunological diseases, further studies are needed to elucidate the precise functions and underlying molecular mechanisms in patients and *in vivo* models.

## Author Contributions

KN and TK conceptualized and conceived the manuscript. KN drafted the manuscript including literature search, reading and writing. KN and TK edited and critically evaluated the manuscript. All authors contributed to the article and approved the submitted version.

## Funding

This work was supported by the Kishimoto Foundation and the Advanced Postdoc Program at the Immunology Frontier Research Center, Osaka University, Japan.

## Conflict of Interest

The authors declare that the research was conducted in the absence of any commercial or financial relationships that could be construed as a potential conflict of interest.

## Publisher’s Note

All claims expressed in this article are solely those of the authors and do not necessarily represent those of their affiliated organizations, or those of the publisher, the editors and the reviewers. Any product that may be evaluated in this article, or claim that may be made by its manufacturer, is not guaranteed or endorsed by the publisher.

## Glossary

**Table:**

3’ UTR	3′ untranslated region
ALI	acute lung injury
Arid	AT-rich interactive domain
βAR	β-adrenergic receptor
C/EBPβ	CCAAT-enhancer-binding proteinβ
CGGA	Chinese Glioma Genome Atlas
CNS	central nervous system
CPZ	chlorpromazine
CRC	colorectal cancer
CTLA-4	cytotoxic T lymphocyte-associated protein 4
DHT	dihydrotestosterone
EAE	experimental autoimmune encephalomyelitis
eIF2a	eukaryotic translation initiation factor 2a
EMT	epithelial-to-mesenchymal transition
ER	estrogen receptor
Erk	extracellular-regulated kinase
Fosl2	fos-like antigen 2
GBM	glioblastoma multiform
GCN2	general control nonderepressible 2 kinase
HCMV	human cytomegalovirus
IDH	dehydrogenase
IL6	interleukin 6
LGGs	low-grade gliomas
LncRNA	long noncoding RNA
LPS	lipopolysaccharide
MDSCs	myeloid-derived suppressor cells
MEFs	mouse embryonic fibroblasts
Mek	mitogen-activated protein kinase kinase
mRNA	messenger RNA
MS	multiple sclerosis
NFκB	nuclear factor kappa-light-chain-enhancer of activated B cells
OxPAPC	oxidized 1-palmitoyl-2-arachidonoyl-snglycero-3-phosphocholine
PCa	Prostate cancer
PDAC	pancreatic ductal adenocarcinoma
PSA	prostate-specific antigen
RA	rheumatoid arthritis
RBPs	RNA-binding proteins
ROS	reactive oxygen species
STAT	signal transducer and activator of transcription
TAMs	tumor-associated macrophages
TFs	transcription factors
TME	tumor microenvironment
TNFα	tumor necrosis factor α
Tregs	regulatory T cells
Trib3	tribbles homolog 3
TRIF	TRIF Toll interleukin-1 receptor homology domain–containing adaptor inducing IFN-β
